# The Static Standing Postural Stability Measured by Average Entropy

**DOI:** 10.3390/e21121210

**Published:** 2019-12-10

**Authors:** Sung-Yang Wei, Chang Francis Hsu, Yun-Ju Lee, Long Hsu, Sien Chi

**Affiliations:** 1 Department of Electrophyics, National Chiao Tung University, Hsinchu 30010, Taiwan; mejicomejico@yahoo.com.tw (S.-Y.W.); francis-920@hotmail.com (C.F.H.); long@cc.nctu.edu.tw (L.H.); 2 Department of Industrial Engineering and Engineering Management, National Tsing-Hua University, Hsinchu 30013, Taiwan; yunjulee@ie.nthu.edu.tw; 3 Department of Photonics, National Chiao Tung University, Hsinchu 30010, Taiwan

**Keywords:** center of pressure (COP), average entropy (AE), entropy of entropy (EoE), multiscale entropy (MSE), inverted U curve, biological disorder, biological complexity

## Abstract

Static standing postural stability has been measured by multiscale entropy (MSE), which is used to measure complexity. In this study, we used the average entropy (AE) to measure the static standing postural stability, as AE is a good measure of disorder. The center of pressure (COP) trajectories were collected from 11 subjects under four kinds of balance conditions, from stable to unstable: bipedal with open eyes, bipedal with closed eyes, unipedal with open eyes, and unipedal with closed eyes. The AE, entropy of entropy (EoE), and MSE methods were used to analyze these COP data, and EoE was found to be a good measure of complexity. The AE of the 11 subjects sequentially increased by 100% as the balance conditions progressed from stable to unstable, but the results of EoE and MSE did not follow this trend. Therefore, AE, rather than EoE or MSE, is a good measure of static standing postural stability. Furthermore, the comparison of EoE and AE plots exhibited an inverted U curve, which is another example of a complexity versus disorder inverted U curve.

## 1. Introduction

Postural stability is a major public health concern in modern society. Based on a World Health Organization (WHO) global report [1], approximately 28%–35% of people aged of 65 years and over fall each year, and this rate increases to 32%–42% for those over 70 years of age. The average cost of hospitalization for instability-related injuries for people 65 years and older in the United States is projected to increase to US $240 billion by 2040. Accurate identification of individual postural instability factors, including impaired balance and poor vision, can greatly increase the likelihood of selecting an appropriate prevention or treatment strategy that is targeted to meet the needs of the individual person [1].

Maintaining postural stability involves a complex sensorimotor control system in the human body [2]. Any disturbance from the surroundings or subjects’ breathing could cause an increase in static standing postural instability [3], and such a loss of static standing postural stability can be shown in the center of pressure (COP) or the center of foot pressure (CFP) trajectory [4,5,6]. Force platforms are simple devices that can be used to record subjects’ COP trajectory over time in anteroposterior (AP) and mediolateral (ML) directions [7]. Open eyes (denoted here as “O”) or closed eyes (denoted here as “C”) are some of the simplest variables that influence subjects’ static standing postural stability [8], since vision can provide a great amount of information regarding postural stability [9]. Further, unipedal (denoted here as “1”) and bipedal (denoted here as “2”) standing also influence subjects’ postural stability [10]. Since leg muscles consume more energy in unipedal than in bipedal conditions [11], postural stability shown by COP data in unipedal conditions is lower. Combinations of these four variances (closed eyes, open eyes, unipedal, and bipedal) are also used for balance testing [12], assistive device evaluations [13], or balance control training [14]. The trend of postural instability, from the most stable to the most unstable conditions, goes from bipedal with open eyes (O2), to bipedal with closed eyes (C2), to unipedal with open eyes (O1), and to unipedal with closed eyes (C1).

There are some entropy-based methods, such as the multiscale entropy (MSE) method, that can be used to distinguish these characteristics under different balance conditions [15,16]. In such studies, higher MSE values are thought to be more stable; for example, MSE complexity index (CI) values under open eyes conditions are usually larger than those under closed eyes conditions [16]. However, there are still some issues that require further discussion. For example, larger MSE CI values are not always more stable. Subjects who exhibit larger COP trajectory variances, which are more unstable, are generally associated with greater MSE CI values, and vice versa [17]. Further, due to the use of different data processes in these studies, the MSE CI values for the closed eyes groups are sometimes higher than those of open eyes groups [15,18].

Recently, average entropy (AE) has been proposed to measure the disorder of biological time series [19]. Heart rate time series signals from atrial fibrillation (AF) groups, which are unhealthy, are more disordered than those from healthy groups. Therefore, the AE method can be used to measure the disorder of heart rate time series from different groups. The entropy of entropy (EoE) method has been proposed to measure the complexity of biological time series [20]. Heart rate signals from healthy groups are more complex or have larger EoE values than those from AF groups. 

In this study, the AE, EoE, and MSE methods were used to individually measure the disorder and the complexity of 11 subjects’ COP data collected under four different balance conditions to determine which method is suitable to measure static standing postural stability.

## 2. Materials and Methods

### 2.1. Materials and Experiment Setup

The 11 subjects included 2 females and 9 males. Their average age ± standard deviation was 30.27 ± 10.76 years old, their average height ± standard deviation was 170.86 ± 4.93 cm, and their average mass ± standard deviation was 67.06 ± 8.34 kg. Subjects had no ankle or knee injuries; did not suffer from any neurological conditions that might affect their balance, such as vestibular disorders or diabetes; and did not take any medications that affected their balance. The project was approved by the National Tsing Hua University Institutional Review Board, and all subjects provided written informed consent before taking part in the experimental procedures. The project identification code was REC10710HE070 (NTHU) (approval date: 30 November 2018).

Subjects were asked to stand on the force platform under four kinds of balance conditions for 30 s, and the data in the first and the last two seconds were removed. Subjects were asked to stand as still as possible in the center of the force platform without locking their knee joints and with their arms relaxed at their sides [21]. For the bipedal tests, the spaces between subjects’ two legs were as wide as their shoulders and symmetric to the center of the force platform. For the unipedal test, the subjects were asked to use the leg on the same side as their dominant hand and to stand on the center of the force platform. Under the open eyes conditions, subjects were asked to stare at a point two meters in front of them. Under the closed eyes conditions, they were asked to close their eyes once they were standing on the force platform.

### 2.2. Average Entropy (AE) and Entropy of Entropy (EoE) Analyses

The algorithms of both AE [19] and EoE [20] methods consist of three steps in analyzing a time series {*x_i_*} = {*x*_1_, …, *x_N_*} of length *N*. The first and the second steps for these two methods are the same. First, the time series is divided into many consecutive non-overlapping windows of equal length *τ*, *w_j_*^(^*^τ^*^)^ = {*x*_(*j*−1)_*_τ_*_+1_, …, *x*_(*j*−1)_*_τ_*_+_*_τ_* }, where *j* is the window index ranging from 1 to *N*/*τ*. Also, *τ* corresponds to the scale factor.

Second, the Shannon entropy value of each window *w_j_*^(^*^τ^*^)^ is calculated. Suppose that *x_max_* and *x_min_* are the maximum and minimum of all data collected in this study. The range from *x_max_* to *x_min_* is divided into *s*_1_ slices of equal width Δ*s*_1_ = (*x_max_* − *x_min_*)/*s*_1_. Subsequently, the Shannon entropy value *y_j_*^(^*^τ^*^)^ of window *w_j_*^(^*^τ^*^)^ is

(1) $$y_{j}^{\left(\tau \right)}=-\sum_{k=1}^{s_{1}}p_{jk}\;ln\;p_{jk},$$

where *k* ranges from 1 to *s*_1_ and *p_jk_* is the probability to find a certain data point in slice index *k* from window *w_j_*^(^*^τ^*^)^. Repeating the same process for every window results in a Shannon entropy sequence {*y_j_*^{^*^τ^*^}^} of length *N*/*τ*.

Third, the AE value is defined as the average of the Shannon entropy sequence {*y_j_^(^^τ)^*} in the form of

(2) $$\mathrm{AE}\left(\tau \right)=\frac{\sum_{k=1}^{N/\tau }y_{j}^{\left(\tau \right)}}{N/\tau }.$$

On the other hand, the EoE is defined as the Shannon entropy of the Shannon entropy sequence {*y_j_^(^^τ)^*}. The derivation of EoE value is as follows. It can be imagined that all elements of {*y_j_*^{^*^τ^*^}^} distribute over some finite levels. Considering all possible distributions of the elements of {*y_j_*^{^*^τ^*^}^}, we obtain the maximum number of all possible levels *s*_2_ (*τ*), which depends upon the time scale *τ*. For example, *s*_2_ (1) = 1, *s*_2_ (2) = 2, *s*_2_ (3) = 3, *s*_2_ (4) = 5, *s*_2_ (5) = 7, and *s*_2_ (6) = 11. Then, the probability *p_l_* to find a certain element in level *l* from the sequence {*y_j_*^(^*^τ^*^)^} is obtained in the form of

(3) $$p_{l}=\frac{total\;number\;of\;y_{j}^{\left(\tau \right)}over\;\left\{y_{j}^{\left(\tau \right)}\right\}\;in\;level\;l}{N/\tau },$$

where *l* is the level index from 1 to *s*_2_. Thus, the resulting Shannon entropy value of the sequence {*y_j_*^(^*^τ^*^)^}, referred to as the EoE value of the original time series {*x_i_*}, is given by

(4) $$\mathrm{EoE}\left(\tau \right)=-\sum_{l=1}^{s_{2}}p_{l}\ln p_{l}.$$

In this study, the AE and EoE methods were used to measure the disorder and the complexity of the COP data of 11 subjects under the four kinds of balance conditions, respectively. The computation was performed using MATLAB (v7, The Mathworks, Inc., Natick, MA, USA) and LabVIEW (2017, National Instrument, Austin, TX, USA). Raw trajectories were extracted from the force platform, the AccuGait System with AMTI’s powerful NetForce/BioAnalysis software package, at a sampling rate of 250 Hz. The extracted data were smoothed with a 4th order Butterworth low-pass filter with a cut-off frequency of 10 Hz. This resulted in two smoothed time series in ML directions $\left\{x_{i}^{ML}\right\}$ and AP directions $\left\{y_{i}^{AP}\right\},$ separately, which lead to an instantaneous COP sway speed time series $\left\{v_{i}\right\}$ according to

(5) $$v_{i}=\frac{\sqrt{{\left|x{\;}_{i+1}^{ML}-x{\;}_{i}^{ML}\right|}^{2}+{\left|y{\;}_{i+1}^{AP}-y{\;}_{i}^{AP}\right|}^{2}}}{1/250},\;\mathrm{for}\;1\leq i\leq L,$$

where *L* = *N* − 1 and *N* is the data size of $\left\{x_{i}^{ML}\right\}$, $\left\{y_{i}^{AP}\right\}$.

Subsequently, the sway speed time series $\left\{v_{i}\right\}$ of each subject was analyzed by the AE and EoE methods. The parameters of $\left(v_{min,\;}v_{max,\;}\;\tau ,s_{1}\right)$ were set at (0 mm/s, 300 mm/s, 5, 300), where *v_min_* and *v_max_* were the minimum and the maximum speed values found in all the sway speed time series data of the 11 subjects, respectively.

### 2.3. An Example of AE and EoE in Analyzing the COP Speed Time Series under Four Kinds of Balance Conditions

**Figure 1:**
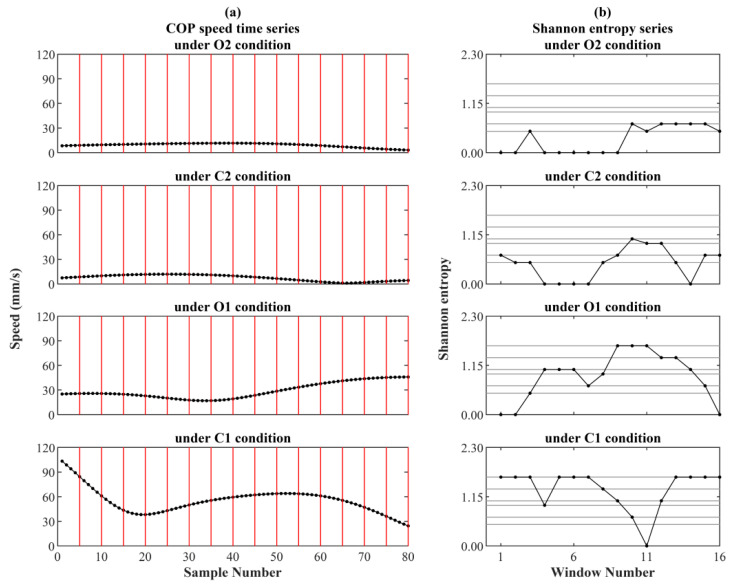
An application of the average entropy (AE) and entropy of entropy (EoE) methods. (**a**) The four original center of pressure (COP) speed time series {*v_i_*} recorded from a certain subject under the four kinds of balance conditions, separately. Each series was equally divided into 16 windows with each of 5 data points in a red frame. The Shannon entropy value of the 5 data points in each window in red was calculated individually. (**b**) The four sets of Shannon entropy sequences {*y_j_*^(5)^} with each of the 16 elements, separately.

Figure 1 demonstrates an application example of the AE and EoE methods. Figure 1a shows the four sets of COP speed time series {*v_i_*} recorded from a certain subject under the four kinds of balance conditions, separately. For simplicity, all series were analyzed with an equal length of *L* = 80 at *τ* = 5. Each series was equally divided into 16 (= *N*/*τ*) windows with each of 5 data points in a red frame. The Shannon entropy value of each window in red was calculated individually. Figure 1b shows the four sets of Shannon entropy sequences {*y_j_*^(5)^} with each of the 16 elements, separately. Then, the average and the Shannon entropy of each of the four Shannon entropy sequences were the AE and EoE values of each COP speed time series, separately. Table 1 lists the AE and EoE values of the four series {*v_i_*} with the AE values in ascending order. It can be seen that EoE is maximal under the O1 condition.

### 2.4. Multiscale Entropy Method (MSE) Analysis

MSE is a widely used complexity measure for physiologic signals. The algorithm of the MSE analysis method of a time series consists of three steps [15,16]. Like the first step of the AE and EoE methods, the first step of MSE method is to divide a time series {*x_i_*} into *N*/*τ* consecutive and non-overlapping windows *w_j_*^(^*^τ^*^)^ with each of equal length τ. The second step is to calculate the average value of the τ elements in each window according to

(6) $$y_{j}^{\left(\tau \right)}=\frac{1}{\tau }\;\sum_{i=\left(j-1\right)\tau +1}^{j\tau }\;x_{i},\;\;1\leq j\leq N/\tau ,$$

Thus, all the average values of the *N*/*τ* windows form a new coarse-grained time series {*y^(^^τ)^_j_*}.

The third step is to calculate the MSE value of the original series {*x_i_*} as the sample entropy of the coarse-grained time series {*y^(^^τ)^_j_*} as follows. Consider the *m*-length vectors $u_{m}\left(p\right)=\;\left\{y_{p}^{\left(\tau \right)},\;y_{p+1}^{\left(\tau \right)},\ldots ,\;y_{p+m-1}^{\left(\tau \right)}\right\},\;\mathrm{where}\;1\leq p\leq N/\tau -m+1.$ Another vector *u_m_* (*q* ≠ *p*) is considered similar to *u_m_* (*p*) if the following equation is satisfied

(7) $$\max\left\{\left|y_{p+k}^{\left(\tau \right)}-y_{q+k}^{\left(\tau \right)}\right|:0\leq k\leq m-1\right\}\leq r$$

Then, set *n_p_^m^* (*r*) as the number of vectors *u_m_* (*q* ≠ *p*) which are similar to the template vector *u_m_* (*p*). The MSE value of the original time series {*x_i_*} is given by

(8) $$\mathrm{MSE}\;\left(\tau \right)=\ln\frac{\sum_{p}^{N-m}n_{p}^{m}}{\sum_{p}^{N-m}n_{p}^{m+1}}.$$

In the following analysis, the parameters (*m*, *r*) for were set at (2, 0.15 × *SD*) where *SD* is the standard deviation of the original time series as suggested [15,16].

In this study, the MSE method was also used to analyze the COP data under the four kinds of balance conditions. Two kinds of data pre-processing were performed for comparison. The result of the first kind was used to compare with those of the AE and EoE methods. In the original MSE study of heart rate analysis, MSE values at τ =20 for the healthy heart rate time series were shown to be significantly higher than those of two pathologic groups, and the classification performance for MSE values at scale 20 was the best when compared to MSE values at other scales. Thus, the scale τ= 20 of MSE method was set [22].

The other kind of data pre-processing method of the MSE method followed the previous study for best discrimination of postural steadiness [15]. Raw trajectories were extracted from the force platforms in both the AP and the ML directions with a sampling rate of 250 Hz. To filter noise and to pick up the frequency intervals of interest, the COP time series were detrended with the ensemble empirical mode decomposition (EEMD) method before the MSE analysis. The ensemble number for the EEMD was 100. All intrinsic mode functions (IMFs) were the averages of these 100 times EEMD results. Twelve unique IMF combinations were generated and IMFs 8–12 were removed, which exhibited frequencies below 0.2 Hz, to ensure a minimum number of dynamic patterns within the length of our time series. Additionally, IMFs 1 and 2 were also removed, as they exhibited frequencies above 20 Hz and were thus unlikely to reflect balance-related biological processes. The continuously sequenced combinations of IMFs 3–6 were used in the AP direction and IMFs 3–7 were used in the ML direction for the best to distinguish each sway direction. The details of the characteristic frequencies of IMFs are shown in Table 2. Then, the MSE complexity index (CI) was used to measure the complexity of the COP trajectory time series [15]. In this study, the MSE^AP^ CI and MSE^ML^ CI in AP and ML directions were set as

(9) $${\mathrm{MSE}}^{AP}\mathrm{CI}\;=\overset{25\;}{\underset{\tau =1}{\sum }}{\mathrm{MSE}}^{AP}\left(\tau \right)$$

and

(10) $${\mathrm{MSE}}^{ML}\mathrm{CI}\;=\sum_{\tau =1}^{35\;}{\mathrm{MSE}}^{ML}\left(\tau \right),$$

respectively.

**Figure 2:**
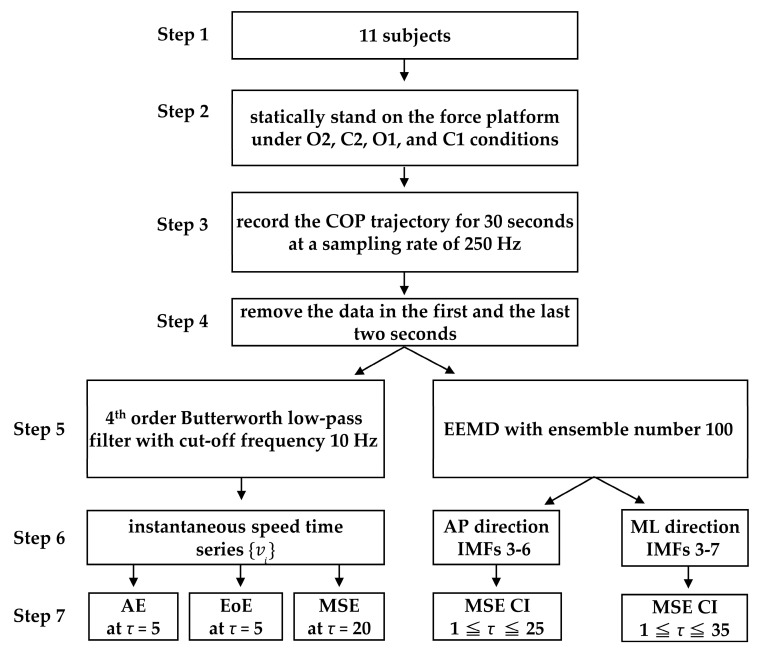
Analytical procedure flowchart in this study.

Figure 2 summarizes the analytical procedure flow chart in this study.

## 3. Results

**Figure 3:**
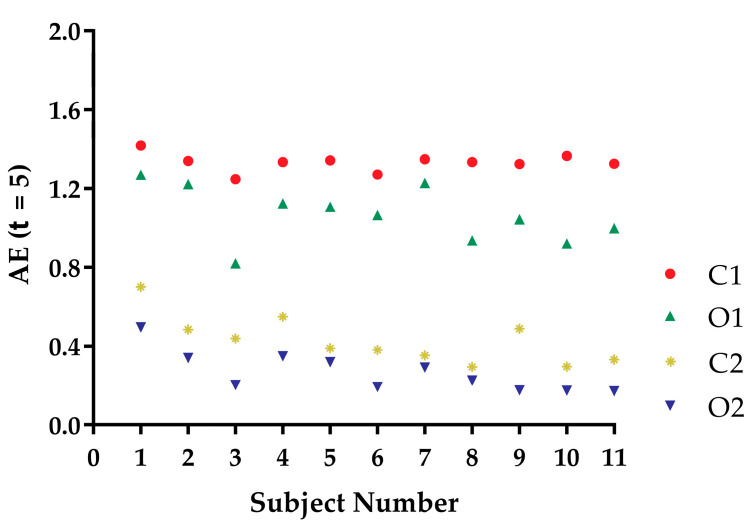
AE values of the COP speed time series from the 11 subjects under four kinds of balance conditions. Recall that the abbreviations of conditions O2, C2, O1, and C1 stood for bipedal with open eyes, bipedal with closed eyes, unipedal with open eyes, and unipedal with closed eyes, respectively. The symbols of blue triangles, yellow stars, green triangles, and red circles represent O2, C2, O1, and C1 conditions, respectively.

Figure 3 shows the AE (τ=5) values of the COP speed time series from the 11 subjects under the four kinds of balance conditions from stable to unstable: O2, C2, O1, and C1. As shown in Figure 3, 100% of the AE results had the following trend: AE (O2) < AE (C2) < AE (O1) < AE (C1); that is, the lower the AE values, the more stable the static standing. 

In a previous study, AE was shown to be a measure of disorder [19]. In this study, disorder was considered to be the same as postural instability. Based on the trends of the subjects (Figure 3), the AE method was demonstrated to be a good measure of static standing postural instability of the COP speed time series under different balance conditions.

**Figure 4:**
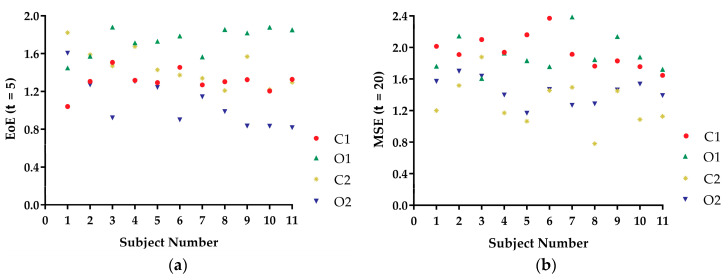
(**a**) EoE values and (**b**) MSE values of the COP speed time series from the 11 subjects under four kinds of balance conditions: O2, C2, O1, and C1.

Figure 4a shows the EoE (τ= 5) values and Figure 4b shows the MSE (τ= 20) values of the COP speed time series from the 11 subjects under the four kinds of balance conditions. The O1 conditions had the largest EoE values for most of the 11 subjects. All the trends of the EoE values and the MSE values were different across subjects and did not follow the postural instability trend. Since both the EoE values and the MSE values are measure of complexity [20,22], the results suggested that complexity could not reflect static standing postural stability. 

**Figure 5:**
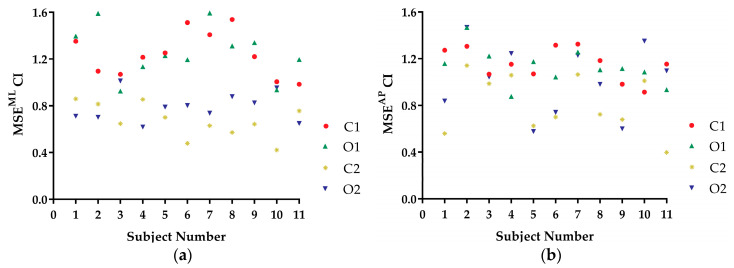
MSE complexity index (CI) values of COP trajectories time series in (**a**) mediolateral (ML) direction and (**b**) anteroposterior (AP) directions from the 11 subjects under four kinds of balance conditions: O2, C2, O1, and C1.

Figure 5a,b shows the MSE CI values of the COP trajectory time series from the 11 subjects under the four kinds of balance conditions in the ML direction and in the AP direction, respectively. Only 1 of the 11 subjects (subject 4) shown in Figure 5a followed the postural instability trend of MSE CI (O2) < MSE CI (C2) < MSE CI (O1) < MSE CI (C1). None of the 11 subjects shown in Figure 5b followed this trend.

MSE CI values are considered to be a measure of complexity [15,16]. In this study, the MSE CI values could not measure static standing postural stability. Based on the trends of the subjects (Figure 5a,b), the MSE method cannot be considered a good measure of static standing postural instability of COP trajectory time series under different balance conditions.

**Figure 6:**
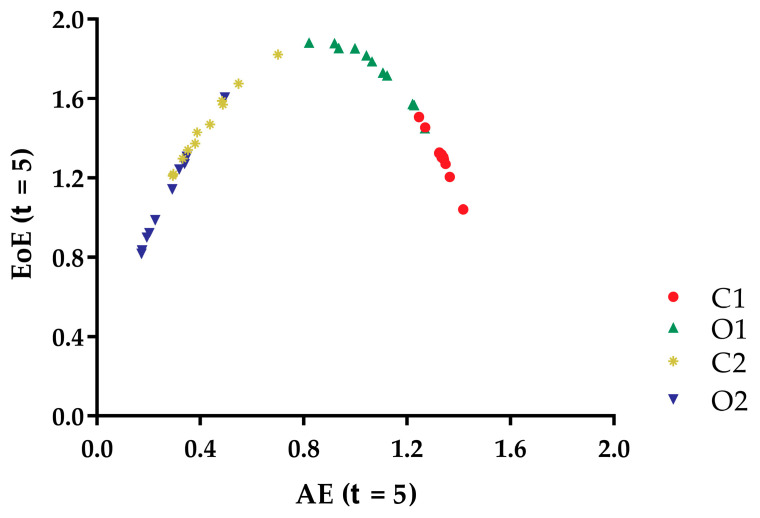
The EoE versus AE of the 44 COP speed time series from the 11 subjects under four kinds of balance conditions: O2, C2, O1, and C1.

Figure 6 shows the EoE versus AE of the 44 COP speed time series from the 11 subjects under four kinds of balance conditions. The plot exhibited an inverted U curve, where the maximal complexity value appears in the O1 conditions, between the largest and smallest AE values.

This inverted U relation exhibited in the complexity (EoE value) versus the disorder (AE value) plot of the COP speed time series in another example of an inverted U relation.

## 4. Discussion

AE measures the disorder of a time series, while EoE and MSE measures the complexity of that. In Figure 3, the AE values of the COP speed series for a certain subject under O2, C2, O1, and C1 conditions were arranged in ascending order. The result was consistent with our intuition of the static standing postural instability. For comparison, EoE was maximal for series under O1 conditions for most of the subjects as shown in Figure 4. Thus the plot of AE vs. EoE exhibited an inverted U shape, as can be seen in Figure 6. However, neither EoE nor MSE of the COP series had distinct trends under O2, C2, O1, and C1 conditions in Figure 4 and Figure 5. The result supported the idea that disorder of the COP speed series can reflect the static standing postural stability better than complexity of the series.

To our knowledge, the idea of the inverted U relation was first conceptually proposed by Huberman and Hogg in 1986 [23]. In 2017, we proposed EoE analysis [20] to measure the complexity of a physiologic time series and applied it to analyze heart rate time series from healthy subjects, patients with congestive heart failure (CHF), and patients with atrial fibrillation (AF). For heart rate time series, the diseased systems suffering CHF exhibited relatively regular behavior. On the contrary, the AF systems exhibited highly erratic fluctuations with statistical properties resembling uncorrelated noise. As a result, EoE robustly separated the healthy group and pathologic groups with AF and CHF by assigning the highest complexity value to the healthy group. The plot of complexity characterized by EoE and disorder characterized by the Shannon entropy of heart rate time series exhibited an inverted U relation. In 2019, we proposed AE as a measure of disorder of time series [19]. We demonstrated that the plot of complexity measured by EoE versus the disorder measured by AE of heart rate time series exhibited a distinct inverted U relation [19].

Different from the heart rate variability, the balance stability can be affected by the balance control systems including visual, the muscles on limbs, and the plantar cutaneous afferent information [9,11,13]. Comparing posture in darkness and posture with full-field vision revealed a significant increase in stability with vision [9]. On the other hand, efficient postural control is important in maintaining balance stability. Standing under unipedal conditions is inefficient for postural control, due to the muscles on limbs requiring more energy expenditure for balance [11]. There were also studies that showed that the increased plantar cutaneous afferent information due to bipedal conditions was able to compensate for deteriorated postural control due to loss of visual systems [13]. Therefore, the trend of postural instability, from the most stable to the most unstable conditions, should be O2, C2, O1, and C1. The better the balance control system performs, the higher the balance stability.

The health condition of the heart can be measured by the complexity of the time series of the heart rate, and this complexity is measured by MSE or EoE. Higher complexity indicates a healthier heart. Static standing postural stability can be measured by the disorder of the time series of the center of pressure of the body on a force platform, and this disorder is measured by AE. Greater disorder indicates unstable static standing. Heart beats are controlled by the autonomic nervous system, but static standing postural stability is controlled by the nonautonomic nervous systems (the somatic nervous system). These facts suggest that disorder measurement is useful in a nonautonomically controlled nervous system, and complexity measurement is useful in an autonomic system. We are conducting further experiments to support these claims.

## 5. Conclusions

We have shown that AE, a measure of disorder, can measure static standing postural stability. For the 11 subjects under four kinds of balance conditions, the AE values of each individual subject increased from stable to unstable conditions. On the other hand, MSE or EoE, as measures of complexity, could not measure static standing postural stability.

Furthermore, the plot of the complexity (EoE) value versus the disorder (AE) value of the COP speed time series of the 11 subjects under four kinds of balance conditions exhibited an inverted U relation. This inverted U relation exhibited in the complexity versus disorder plot of the COP speed time series is another example of an inverted U relation, similar to that which was exhibited for heart rate time series signals.

## Figures and Tables

**Table 1:** The AE and EoE values of the four sets of COP speed time series under four kinds of balance conditions.

Balance Conditions	AE (*τ* = 5)	EoE (*τ* = 5)
O2	0.30	1.02
C2	0.48	1.49
O1	0.91	1.84
C1	1.32	1.25

**Table 2:** Details of the intrinsic mode functions (IMFs) and multiscale entropy (MSE) scales used in this study.

COP Direction	IMFs	Characteristic IMF Frequencies (Hz)	MSE Scales
AP	3, 4, 5, and 6	19.43, 8.40, 3.49, and 1.36	1–25
ML	3, 4, 5, 6, and 7	19.36, 7.79, 3.26, 1.19, and 0.52	1–35
